# Routine handling methods affect behaviour of three-spined sticklebacks in a novel test of anxiety

**DOI:** 10.1016/j.bbr.2016.03.015

**Published:** 2016-06-01

**Authors:** Ralph R.J. Thompson, Elizabeth S. Paul, Andrew N. Radford, Julia Purser, Michael Mendl

**Affiliations:** a Centre for Behavioural Biology, School of Veterinary Science, University of Bristol, UK; b School of Biological Sciences, University of Bristol, UK

**Keywords:** Stickleback, Anxiety, Handling, Scototaxis, Novel tank diving test, Relief

## Abstract

Fish are increasingly popular subjects in behavioural and neurobiological research. It is therefore important that they are housed and handled appropriately to ensure good welfare and reliable scientific findings, and that species-appropriate behavioural tests (e.g. of cognitive/affective states) are developed. Routine handling of captive animals may cause physiological stress responses that lead to anxiety-like states (e.g. increased perception of danger). In fish, these may be particularly pronounced when handling during tank-to-tank transfer involves removal from water into air. Here we develop and use a new combined scototaxis (preference for dark over light areas) and novel-tank-diving test, alongside conventional open-field and novel-object tests, to measure the effects of transferring three-spined sticklebacks (*Gasterosteus aculeatus*) between tanks using a box or net (in and out of water respectively). Preference tests for dark over light areas confirmed the presence of scototaxis in this species. Open-field and novel-object tests failed to detect any significant differences between net and box-handled fish. However, the combined diving and scototaxis detected consistent differences between the treatments. Net-handled fish spent less time on the dark side of the tank, less time in the bottom third, and kept a greater distance from the ‘safe’ bottom dark area than box-handled fish. Possible explanations for this reduction in anxiety-like behaviour in net-handled fish are discussed. The combined diving and scototaxis test may be a sensitive and taxon-appropriate method for measuring anxiety-like states in fish.

## Highlights

• We develop a new combined diving and scototaxis test of anxiety in fish.
• We compare box (in water) and net (out of water) transfer between tanks.
• Net transfer results in less anxiety like-behaviour. Explanations are considered.
• Novel-object and open-field tests fail to detect these differences.
• The combined diving and scototaxis test is a promising biologically-meaningful test.

## 1 Introduction

Recent studies have shown that the way in which laboratory rodents are handled may profoundly influence anxiety-like behaviour with potential knock-on effects for welfare and experimental outcomes [1]. Fish are increasingly important laboratory animals [2], [3], [4], [5] and are frequently ‘handled’ by transferring them between tanks using nets that remove them from water for the duration of transport. This handling method may have welfare and behavioural implications, particularly in laboratories where fish are moved regularly for behavioural testing [6], and has been used as a manipulation in experiments investigating the time-course of physiological stress responses in fish [7], [8], [9].

Whilst fear is hypothesised to occur in response to immediate and present threats, anxiety may occur when an animal perceives increased uncertainty about a potential future threat, or enhanced probability of danger [e.g. 10], for example in the presence of cues that predict danger or uncertain outcomes, or following recent dangerous events (e.g. being caught and removed from the water in the case of an aquatic species). Consequently, tests of exploratory behaviour are often used as measures of animal anxiety, with the prediction that increased anxiety should result in decreased risk-taking and exploration and an increased tendency to remain in ‘safe’ areas—so-called ‘anxiety-like’ behaviour. In fish, examples of such tests include measures of thigmotaxis (the tendency to remain close to walls or other solid objects [11], [12]), open-field use [e.g. 2], novel object inspection [e.g. 13], novel-tank-diving [14], [15], and scototaxis (the preferential movement of an organism to a dark area, as distinct from negative phototaxis [16], [17], [18]).

In their review of the validity of fish models of anxiety, Maximino et al. [19] identified the novel-tank-diving paradigm as having the best-established predictive validity. When fish are transferred to a new tank they usually swim to the bottom, a response that may be adaptive in helping them to avoid any predator lurking above. Fish taking a longer time to move back up towards the surface are assumed to be more anxious, and this is supported by studies showing that this behaviour changes as predicted in response to pharmacological agents that are known to have anxiolytic or anxiogenic effects in humans and other species [e.g. 20].

Maximino et al. [19] also argued that scototaxis has good construct validity (i.e. it ‘makes sense’ in light of fish ecology and evolutionary theory). Scototaxis-based exploratory tests are seen as appropriate measures of anxiety because they present the fish with a motivational conflict between staying in the perceived safest area (the dark area) and exploring the perceived more risky (light) area in order to discover potential resources (food/mates/passage to an even safer place). The light areas of tanks are thought to be more dangerous because they reduce the background-matching camouflage of fish, particularly when seen from above as they would be by an aerial predator.

Pharmacological tests indicate that particular drugs (e.g. chlordiazepoxide; citalopram) may not induce consistently anxiogenic or anxiolytic effects in both the novel-tank-diving and scototaxis tests, suggesting that these tests may reflect slightly different motivational or affective processes [19]. A combination of the novel-tank-diving and scototaxis methods may therefore be a sensitive, novel, and biologically meaningful way of identifying anxiety-like states in fish, and one aim of the current study was to develop such a combined test.

The three-spined stickleback (*Gasterosteus aculeatus*) is an important study species in behavioural and neurobiological research, including in recent investigations into the influence of anthropogenic disturbance on animal behaviour [21], [22], [23], [24], [25], [26]. Brydges et al. [27] investigated the effects of tank-transfer methods in this species and found higher physiological stress responses (as measured by increases in opercular gill beat rate and cortisol) in individuals that had been transferred using a net rather than using a ‘scoop’ which kept the fish in the water. Surprisingly, however, the apparent stress of the net transfer procedure did not translate into subsequent increased anxiety-like behaviour as measured by conventional emergence-latency and novel-object tests. Here, we develop and use a new combined diving and scototaxis test of anxiety-like behaviour to explore further the implications of handling using a net vs. scoop/box method in this species.

The stickleback populations from which our subjects were drawn live in habitats with sharply contrasting dark and light areas due to shadows cast by vegetation on a light toned substrate in clear water (unpublished field observation). Like many British sticklebacks, they are likely to have been under high predation pressure from birds [28], [29], many of which have been recorded in the area from which the fish were sourced [e.g. 30]. Sticklebacks, in common with many fish, have a counter-shaded body [31] with a dark upper and a lighter, reflective lower body, making them appear dark when seen from above thus camouflaging them against dark backgrounds. They also actively adapt their dorsal colouration to improve background matching [32], attesting to the importance of this camouflage. There is thus a good case for expecting scototaxis in sticklebacks. The tendency to stay in relatively safe areas of a tank will, however, be traded-off against motivation to forage and explore [33] and there appears to be no published scientific evidence to support the idea that sticklebacks will prefer to be in an area with a dark background. Thus, one aim of the current study was to establish that such a preference exists in the three-spined stickleback.

Here, we first establish the suitability of using a scototaxis test of anxiety in the three-spined stickleback by investigating whether the species has the predicted preference for dark over light areas, and whether this is maintained even when fish have had prior experience of living in a light-coloured tank (Experiment 1). We then carry out standard open-field and novel-object tests [cf. 27] to investigate the effects of net and scoop/box handling, with the prediction that more anxious individuals will spend more time away from the central area and close to the tank walls, and will be slower to approach the novel object (Experiment 2). In addition, we use both black- and white-walled test tanks to investigate whether any effects of handling are more readily revealed in an apparently more dangerous (white) or less dangerous (black) environment, and also whether anxiety-like behaviour is more clearly observed in the lighter tank. Finally, we combine scototaxis and novel-tank-diving paradigms to create a new test incorporating both principles, and we investigate whether this approach can detect any differential effects of net and scoop/box handling on anxiety-like behaviour (Experiment 3) and, if so, whether it is more sensitive than traditional open-field and novel-object tests. Based on our discussion above, the more time a fish spends in the darker side of the tank, near the bottom, and close to the dark half of the bottom area (putatively the area that is perceived as ‘safest’), the more anxious it is deemed to be.

## 2 Materials and methods

For all experiments, animal husbandry was performed by University animal care staff and researchers at the School of Biological Sciences, University of Bristol. Ethical permission was granted by the University of Bristol Animal Welfare and Ethical Review Body (University Investigation Number: UB/10/020), and procedures complied with the Association for the Study of Animal Behaviour/Animal Behavior Society guidelines for the treatment of animals in behavioural research and teaching [34].

### 2.1 Experiment 1: testing for scototaxis in sticklebacks

#### 2.1.1 Animals and husbandry

Fifty adult three-spined sticklebacks (of mixed, unknown age and sex) were caught using hand-held nets from a freshwater pond in southwest U.K. (51°30′44″N, 2°38′13″W; online still-water associated with Hazel Brook/River Trym) with appropriate Environmental Agency permission. They were transported to the University of Bristol Aquarium Facility and acclimatised to holding tanks (see Ref. [35] for full details). Groups of up to 20 sticklebacks were held in 100-l, white-bottomed, glass tanks (90 × 36.5 cm; water depth: 30 cm) containing artificial plants for shelter, an external power filter and an airstone. Fish were kept in non-breeding condition at 17 °C on a 12:12 h light:dark cycle, and were fed three times weekly with frozen bloodworms (chironomid larvae) and flakes (Aquarian Goldfish Flake, Masterfoods, Batley, U.K.). Tanks were cleaned and water was changed through regular siphon cleaning which was performed slowly and gently, allowing fish to swim out of the way in their own time without manipulation. All fish had been well acclimatised (held in controlled conditions for at least 6 months) prior to the experiment. Following this experiment, fish were retained in the University of Bristol Aquarium Facility for use in other work.

#### 2.1.2 Prior tank experience

##### 2.1.2.1 Black condition

A cohort of 20 of the 50 available animals was moved to a 100-l glass tank with black plastic sheeting surrounding the sides and bottom (open top) two weeks prior to the start of the experiment. Four of these fish died before completion of the experiment, leaving 16 in the experiment. A green and black plastic netted partition was used to divide this tank so that fish that had completed the experiment could be kept separate from naïve ones.

##### 2.1.2.2 White condition

The remaining 30 fish remained in a large (approx. 150 l) opaque walled and bottomed (open topped) white plastic tank until experimentation and were held in a different white-bottomed tank following the experiment to separate fish that had completed the experiment from naive ones.

#### 2.1.3 Apparatus and procedure

**Fig. 1:**
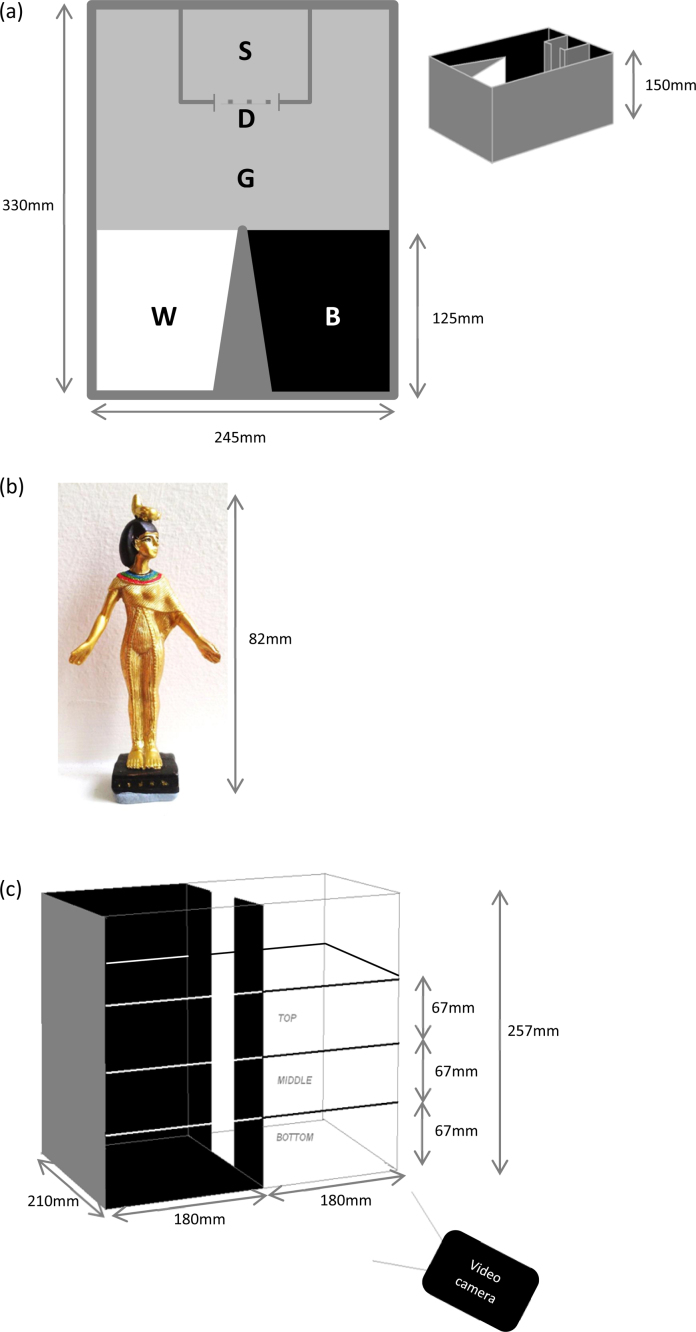
(a) Preference test tank. The fish was placed in the start area (S) and then allowed to swim freely around the entire tank once the perspex door (D) had been opened, with access to the grey (G), white (W) and black (B) areas. Shaded triangle represents a solid, opaque, barrier. (b) Novel object used in the novel-object test. It was an 82 mm tall replica of a statue of Serket found in Tutankhamun’s tomb (purchased from Bristol Museum and Art Gallery). It was mounted on Blue-Tack (Bostik) to secure it to the tank floor when added following the open-field test. (c) The diving/scototaxis test tank was divided in the middle by covered plastic strips, with a gap (250 × 70 mm) between them. One side of the division was back and the other white. The floor and two walls of each side of the tank were also this colour (black or white). The final wall, facing the webcam, was transparent. The three lines on this wall marked the waterline (top line) and divided the water tank in to three equal volumes allowing the observer to see the top, middle and bottom segments of the water. At the start of the experiment, the fish was placed in the top third of the black side of the tank and then allowed to swim freely.

The experiments were carried out in small (approx. 10 l) plastic tanks (preference-test tank) fitted with a transparent perspex start area (with sliding door) facing a wedge-shaped division which separated white- and black-bottomed areas (created with coloured tape). The start box and surrounding areas had a grey bottom and sides (Fig. 1a). Two ‘mirror-image’ tanks were built and used for equal numbers of trials to eliminate the effect of any inherent side bias. Video recordings were made of all trials on a computer using a webcam (Microsoft™ LifeCam, Microsoft Corporation) mounted above the test tank. This was also used to monitor the experiment in real time so that there was no experimenter in the experimental room while the trial was in progress. Test tanks were filled with filtered water with a drop of ‘*Haloex Solution’* added to remove chlorine. Water was stirred between each trial to disperse any chemical cues and completely changed daily.

Fish were tested individually. Each fish was caught from the holding tank using a small net and then added to a transfer jug without removing it from water. It was then transferred to the test tank and released in to the start area with the perspex ‘door’ closed where it was left to acclimate for 2 min. The perspex door was then removed allowing the fish to swim freely around the tank. Using the webcam feed to a computer screen, the position of the centre of the fish’s body (in the start, grey, white or black areas of the tanks) was recorded every 10 s for 10 min (instantaneous sampling: [36]; recorded by RRJT). At the end of this time, the fish was returned to the appropriate post-experimental tank. The number of counts in the black area was divided by the sum of the counts in the two choice areas (black and white) to give a proportion of time spent in the black area out of the total spent in either the black or white area (black/(white + black)). If the fish had no preference between the black and white areas then the expected mean for this value would be 0.5.

### 2.2 Experiment 2: effects of handling methods on behaviour in open-field and novel-object tests

#### 2.2.1 Animals and husbandry

Forty-eight adult three-spined sticklebacks (of mixed, unknown age and sex) were used in this experiment. They were caught using hand held nets from the River Cary at Somerton Door, Somerset, U.K. (O.S. grid reference ST469303) with appropriate Environment Agency permission. They were transported to the University of Bristol Aquarium Facility and acclimatised to holding tanks. They were housed in groups of approximately 100 in two 170 l flecked-grey-bottomed glass tanks containing artificial plants for shelter, an external power filter and an airstone. Fish were kept in non-breeding condition at 15–16 °C on an 8:16 h light:dark cycle and were fed daily with frozen bloodworms (chironomid larvae) and flakes (Aquarian Goldfish Flake, Masterfoods, Batley, U.K.). Tanks were cleaned and water was changed as described in Section 2.1.1. The fish had been handled previously (for group decision-making experiments not discussed here) but not for at least two months prior to testing. Following the experiments, fish were released into a large private pond in University of Bristol grounds (offline still waters) in line with Environment Agency protocol.

#### 2.2.2 Transfer techniques

During experiments, fish were transferred either using a box or a net. Prior to catching the fish, they were first confined in a small area of the housing tank using a large net before being scooped out in the transfer net or box, to avoid chasing the fish around the tank and causing associated stress. The box transfer used a transparent plastic box (34 × 62 × 97 mm) which was placed inside a second box coated in black plastic to make an opaque black water-filled container. The net used was a standard (60 × 75 mm) aquarium net with white mesh and blue metal frame/handle. During experimental transfer the fish were held out of the tank (with net-handled fish out of the water and box-handled fish in water in the box) for 10 s, a period typical of normal husbandry practices and identical to that used by Brydges et al. [27]. Fish were released into the test tanks by submerging, inverting and withdrawing the box or by dragging the net through the surface of the water in such a way that it was inverted releasing the fish into the open water.

#### 2.2.3 Apparatus and procedure

The tests were carried out in rectangular plastic tanks (310 mm × 225 mm; water depth: 60 mm). Two tanks were used, one with white walls and floor and the other with black walls and floor, covered with white or black plastic tape. These were different from the test tank used in Experiment 1. Each had markings on the floor showing bisecting perpendicular lines, the centre point of the tank and the area within 50 mm of the perimeter of the tank. A video camera (Canon Legria FS200 high definition) was fixed c.1 m above the water surface of the tanks to record behaviour during each test.

Sticklebacks were transferred individually using either the box or net method, in a balanced pseudorandom order, and placed in the centre of the open-field tank (either black or white, presented in a pseudorandom order balanced with the order of transfer techniques). The experimenter then left the room and returned 10 min later to place the novel object – a small model statue (see Fig. 1b) – in the centre of the tank, before again leaving the room, returning 5 min later to return the fish to the housing tank. Forty-eight fish were used, 24 in each tank colour and 24 with each transfer method such that 12 were in each of the four (colour × transfer) conditions. Fish were allocated to conditions based on the order in which they were caught, but ensuring that order of allocation to black vs white tanks and net vs box transfer was pseudorandom and counter-balanced. Fish allocated to the different treatments did not differ in length (handling: t = 1.518, df = 35, p = 0.138; tank colour: t = 0.286, df = 35, p = 0.777).

Videos were analysed using EthoVision XT [Noldus Information Technology; [37]] which automatically recorded the position of the fish within the tank 2.5 times per second. This allowed us to generate the following variables for both tests: (i) the mean time that fish spent in a central zone 50 mm away from any of the walls (putatively the most threatening area of the tank); (ii) the mean distance that the fish kept from the central point in the tank (where the novel object was placed in the novel-object test); and (iii) the mean speed of the fish as a measure of general activity. For the novel-object test only, we also analysed: (iv) the latency to enter the central zone after the novel object was placed in the centre of the tank (5 out of 48 fish did not enter this zone during the 5-min tests and their latencies were coded as 300s). Latency to enter the central zone was not measured in the open-field test as fish were placed in the centre of the tank at the start of this test. The tracks of each fish were checked and corrected manually by RRJT before summary data were produced. Variables (i)–(iii) were calculated for each 1-min interval of each test.

### 2.3 Experiment 3: effects of handling methods on behaviour in the combined diving and scototaxis test

#### 2.3.1 Animals and husbandry

Fifty-six three-spined sticklebacks were used in this experiment. They had the same origin and husbandry as those used in Experiment 2, but with no previous handling other than when they were caught from the wild and moved to the laboratory. Following the experiments, fish were released into a large private pond in University of Bristol grounds (offline still waters) in line with Environment Agency protocol.

#### 2.3.2 Apparatus and procedure

A plastic test tank was used (358 mm long × 215 mm wide × 257 mm high; water depth: 205 mm), with three opaque walls and one long transparent wall to allow viewing from the side. The tank was half black and half white (the opaque walls and floor were covered in black/white plastic tape) with a dividing wall coloured white on the white side of the tank and black on the black side, separating the two halves of the tank. A gap (250 × 70 mm) allowed fish to swim between the two sides. The height of the water column was marked into three equal depth zones by lines on the long walls of the tank (Fig. 1c). Trials were recorded in side-view using a Canon Legria FS200 high definition video camera fixed to the side of the test-tank on a shelving unit. The shelving unit also held the test tank and a black plastic surround, which excluded external visual stimuli and prevented reflections from the transparent wall of the test tank (particularly important for automated video tracking of the animals from this side view through the wall of the tank).

Sticklebacks were transferred individually using either the box or net as in Experiment 2, and in a balanced pseudorandom order, to the centre of the top third of the black side of the test tank. The experimenter then left the room and returned 10 min later to stop the recording and return the fish to a housing tank. Twenty-three fish were used in each of the transfer conditions. The mean length of fish in the two treatment groups did not differ (t = 0.688, df = 54, p = 0.494). Videos were analysed using EthoVision XT [Noldus Information Technology; [37]] which automatically recorded the position of the fish within the tank 2.5 times per second allowing us to generate the following variables: (i) the mean time fish spent in the bottom third of the tank; (ii) the mean time they spent in the black side of the tank; (iii) the mean distance they were from the bottom corner of the black side of the tank (putatively perceived as the safest area); and (iv) their mean speed as a measure of general activity. These variables were calculated for each 1-min interval of the test. Data checking was as described for Experiment 2.

### 2.4 Statistical analysis

Data were analysed using IBM SPSS statistics version 21 [38]. Parametric tests (t-tests and general linear models (GLMs)) were used whenever raw or transformed data, or (for GLMs) studentized residuals, were found to be normal by Kolmogorov-Smirnov normality tests and examination of histograms and Q–Q plots. GLMs were initially constructed including all relevant factors (Experiment 2: within-subjects factor: time point in test (in 1-min intervals); between-subjects factors: handling method (box vs net), tank colour (black vs white); Experiment 3: as for Experiment 2 but with no tank colour factor), and interactions. Non-significant factors (p > 0.05) were then sequentially removed to arrive at the minimal model of significant factors which is reported (because of the focus of the study, effects of handling method were always kept in the model). GLM analyses are reported with the Greenhouse-Geisser correction whenever non-sphericity was detected. Partial Eta Squared estimates of effect sizes are given for these factors. Data that did not satisfy parametric testing assumptions, even after transformation, were analysed using Mann-Whitney *U* tests to investigate the effects of handling (and tank colour) on grand-means across the whole test period, and Friedman tests to assess the effects of time point in test.

Variables recorded from the same tests were selected to provide measures that did not inevitably co-vary (e.g. speed and location), or that captured responses to different features of the test (e.g. time spent at the bottom or on the black side of the diving/scototaxis tank). Nevertheless, some variables taken from the same tests may not have been completely independent. To investigate this issue, we performed PCAs on the variables recorded in each test at each time point of that test. Loadings of variables on the resulting principal components (PCs) changed from minute to minute, as did the number of PCs with eigenvalues greater than 1. This indicates that measures within each test were not consistently correlated across time, and therefore that we could not use the PCs and associated factor scores to represent stable underlying factor/s at each time point. Consequently, we analysed variables separately, but applied a sequential Bonferroni correction to the significance level for acceptance of any effects of handling or tank colour. For example, using p < 0.05 and with four variables analysed, the smallest p value resulting from tests of handling or tank colour effects would need to be <0.0125 (0.05/4) to be accepted as significant, the next smallest <0.017 (0.05/3), and so on [39].

## 3 Results

### 3.1 Experiment 1: testing for scototaxis in sticklebacks

**Fig. 2:**
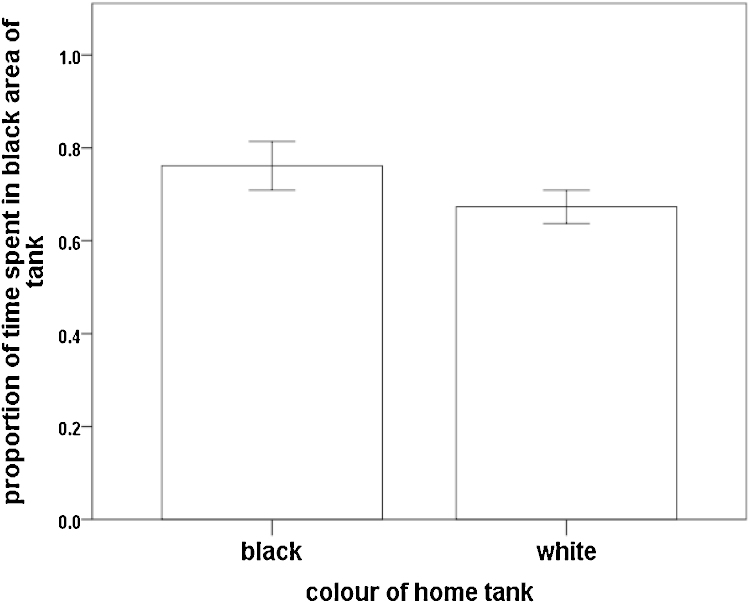
Mean proportion of time (±SEM) spent in the black side of the tank during the preference test (counts in black side divided by the sum of the counts in black and white sides) for fish previously housed in black and white tanks.

When introduced to the test tank (Fig. 1a), fish that had been kept in black housing conditions spent a mean of 4.2 counts in the white area and 14.1 counts in the black area, whilst those that had been kept in white housing conditions spent a mean of 6.4 counts in the white area and 13.5 counts in the black area. Fish housed in both black (one-sample *t*-test: t_15_ = 4.98, p < 0.001) and white (t_29_ = 4.81, p < 0.001) conditions showed a significant preference for the black area of the test tank relative to the white area (combined: t_45_ = 6.78, p < 0.001; Fig. 2). The mean preference for the black area did not differ between fish from black housing and those from white housing (two-sample *t*-test: t_44_ = 1.41, p = 0.164).

### 3.2 Experiment 2: effects of handling methods on behaviour in open-field and novel-object tests

#### 3.2.1 Open-field test

**Fig. 3:**
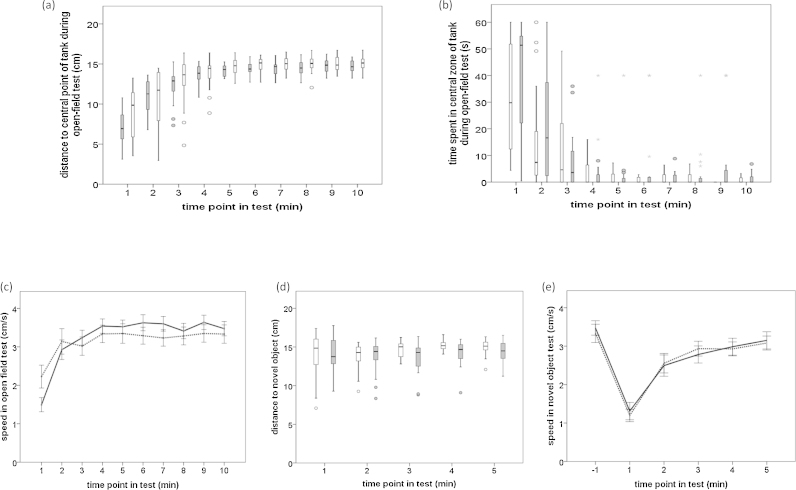
Mean and median responses of net- and box-handled fish per minute of test for open-field test measures of (a) distance to central point of the tank, (b) time spent in central zone of the tank, (c) speed, and for novel-object test measures of (d) distance to novel object and (e) speed. Line graphs show means (±SEM) for data analysed using parametric statistics with net-handled fish being represented by solid lines and box-handled fish by dashed lines. Box-plots show medians, quartiles and ranges for data analysed using non-parametric statistics with (a) fish tested in white tanks represented by white boxes and those tested in black tanks by grey boxes, and (b,d) net-handled fish represented by grey boxes and box-handled fish by white boxes. Data points are indicated if they are greater than 1.5 (circle) or 3 (asterisk) inter-quartile ranges away from the upper or lower quartile.

Because only speed of movement data were normally distributed, Mann-Whitney *U* tests were carried out on grand-means of the other variables, and their results are presented in Table 1. There were no significant effects of handling method or tank colour on duration spent in the central zone of the tank. Mean distance to the central point of the tank was not significantly affected by handling but differed between the two tank colours (p = 0.045, Fig. 3a) with fish in white tanks keeping further away from the central point. However, this was not significant when a sequential Bonferroni correction was applied. To investigate whether handling effects were differentially revealed in white or black tanks, separate analyses were carried out for each tank colour, but no significant effects of handling were observed in either white or black tanks. During the test, distance to the centre point of the tank increased significantly with time (Friedman chi-square = 275.2, df = 9, p < 0.001; Fig. 3a), and duration spent in the central zone of the tank decreased significantly across time (Friedman chi-square = 186.4, df = 9, p < 0.001, Fig. 3b). Mean speed in the open-field test was significantly affected by time (F_3.13,144.13_ = 28.52, p < 0.001, partial Eta-squared = 0.383) and there was an interaction between time and handling (F_3.13,144.13_ = 3.21, p = 0.023, partial Eta-squared = 0.065; Fig. 3c) which appeared to reflect net-handled fish moving slower than box-handled fish during the first minute of the test, but faster later on. However, this was not significant when a sequential Bonferroni correction was applied. There were no significant main or interaction effects of tank colour (p > 0.7) and hence this factor was not included in the final model.

#### 3.2.2 Novel-object test

Table 1 shows results of the Mann-Whitney *U* tests. None of duration in the central zone of the tank, mean distance from the centre of the tank, or latency to enter the central zone of the tank were significantly affected by tank colour or handling method. Net-handled fish had a lower mean distance to the novel object than box-handled fish when tested in the black tank (p = 0.017), but this was not significant after a sequential Bonferonni correction. During the test, duration in the central zone of the tank showed a non-significant tendency to decrease with time (Friedman chi-square = 9.45, df = 4, p = 0.051), and distance to the novel object increased significantly with time (Friedman chi-square = 16.8, df = 4, p = 0.002) although this appeared to be a very subtle change (Fig. 3d). Fish significantly decreased their speed of movement in response to introduction of the novel object (F_1,47_ = 160.26, p < 0.001, partial Eta-squared = 0.773; Fig. 3e: change between −1 and 1 min). Speed then increased significantly during the 5-min of the novel-object test (F_2.17,101.83_ = 59.14, p < 0.001, partial Eta-squared = 0.557; Fig. 3e). Tank colour and handling method did not significantly affect speed in either analysis (p > 0.5 during mins −1 to 1; p > 0.3 during 1–5 min) and so were not included in the final models.

### 3.3 Experiment 3: effects of handling methods on behaviour in the combined diving and scototaxis test

**Fig. 4:**
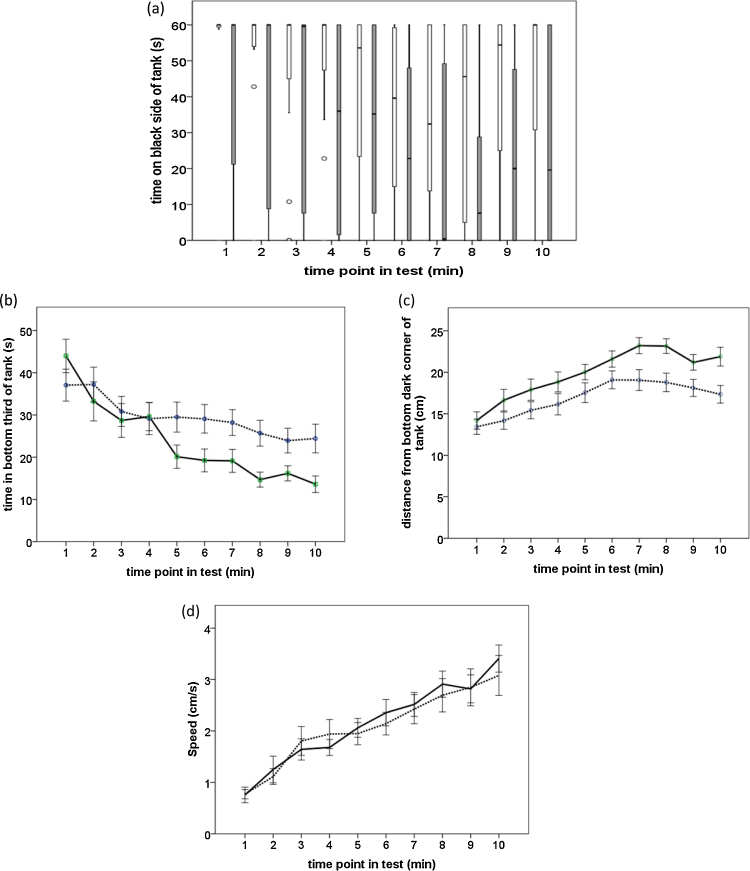
Mean and median responses of net- and box-handled fish per minute of the combined diving and scototaxis test for measures of (a) time spent on the black side of the tank, (b) time spent in the bottom third of the tank, (c) distance from the bottom dark area of the tank, and (d) speed. Line graphs show means (±SEM) for data analysed using parametric statistics with net-handled fish being represented by solid lines and box-handled fish by dashed lines. Box-plots show medians, quartiles and ranges for data analysed using non-parametric statistics, with net-handled fish being represented by grey boxes and box-handled fish by white boxes. Data points are indicated by circles if they are greater than 1.5 inter-quartile-ranges away from the upper or lower quartile.

As Experiment 1 confirmed the presence of scototaxis in the three-spined stickleback, we developed a combined test of scototaxis and novel-tank-diving behaviour, and investigated the effects of handling method on behaviour in this test. Net-handled fish spent significantly less time on the black side of the test tank, and hence more time on the white side, than box-handled fish during the whole test (Mann-Whitney U = 235, n = 56, p = 0.010; Fig. 4a). In addition, time spent on the black side decreased significantly as the test proceeded, though it tended to increase again towards the end of the test (Friedman chi square = 69.73, df = 9, p < 0.001; Fig. 4a). Table 2 summarises the GLM results and shows that there was a significant time*treatment interaction on duration spent in the bottom third of the tank (p = 0.019) reflecting less time spent by the net-handled fish at the bottom of the tank during the second half of the test (Fig. 4b). Post-hoc *t*-tests indicate that treatment groups differed at p < 0.05 for each minute from and including the fifth minute of the test. A sequential Bonferonni correction for 10 post-hoc tests yielded one significant difference at 8 min (p = 0.003). Net-handled fish also showed a significantly higher mean displacement from the bottom black area of the tank throughout the test (p = 0.009; Fig. 4c). Mean speed of movement, however, was not significantly affected by handling treatment (p > 0.4). All of the variables in Table 2 also showed strongly significant changes over time (p < 0.001), consistent with the fish recovering from the effects of the handling and becoming familiar with their new surroundings. Time spent at the bottom of the tank decreased across time (Fig. 4b), whilst displacement from the bottom black area increased (Fig. 4c). Movement speed increased across time from low levels of movement (e.g. freezing/immobility) at the start of the test to faster speeds later on (Fig. 4d).

## 4 Discussion

Experiment 1 showed that sticklebacks prefer areas with a black floor to those with a white floor and hence exhibit the scototaxis response. There was no significant difference in scototaxis between fish housed in the darker and lighter tanks, indicating that sticklebacks are predisposed to show the response irrespective of previous experience. These results are consistent with theoretical predictions that sticklebacks are likely to be in a state of lower perceived danger when they are against a dark background, and hence to behave less anxiously [18]. On this basis, we combined measures of scototaxis with measures of diving behaviour to generate the novel test of anxiety-like states employed in Experiment 3.

There were no significant effects of tank colour or handling treatment on behaviour of the fish in the conventional open-field and novel-object tests used in Experiment 2. This is in line with the findings of Brydges et al. [27] who, despite demonstrating stronger cortisol stress responses in net-handled compared to scoop-handled three-spined sticklebacks, failed to detect any differences in their behaviour in open-field and emergence-latency tests of anxiety behaviour. By contrast, the combined diving and scototaxis test used in Experiment 3 clearly discriminated between the two handling methods. Net-handled fish spent less time on the black side of the test tank, less time in the bottom third of the tank in the second half of the test, and kept a greater mean distance from the safe bottom dark area of the tank than box-handled fish. There were no differences in speed of movement.

Following our definition of anxious fish as those that spend more time in the putatively safer lower portion of a novel tank and against a black background (see Introduction), these findings indicate that net handling, somewhat counter-intuitively, induced lower anxiety than box-handling. Previous work [27] showed that sticklebacks exhibit a greater physiological stress response following handling with a net than with a scoop. Combined with our findings, this suggests a negative relationship between acute physiological stress and subsequent behavioural indicators of anxiety. High levels of physiological stress can lead to more risky behaviour [40] and elevated cortisol can lead to a decrease in punishment-related behaviour and an increase in reward-related behaviour [41], with cortisol effectively appearing to act as an anxiolytic [42], [43], [44]. The physiological stress resulting from net handling could therefore have shifted the trade-off between reducing predation risk (a punishment-related behaviour) and increasing exploration (a reward-related behaviour) in favour of exploration, promoting the observed greater use of the white and top areas of the diving/scototaxis tank.

A different explanation is that the physiological stress response interfered with appropriate decision-making. For example, Purser and Radford [22] showed that noise could impede appropriate decisions by three-spined sticklebacks to attack food vs non-food items, perhaps by interfering with attention processes. Sneddon et al. [45] found that a noxious stimulus reduced neophobic behaviour in response to a novel object in rainbow trout (*Oncorhynchus mykiss)*, and suggested that negative stimuli could thus distract the attention of fish and impede adaptive neophobic responses. Stress-induced shifts in attention away from a task [46], [47] might also underlie the findings here, with fish not attending to the risks of being on the white side or near the water surface, due to the stress induced by net transfer. Further experiments are needed to distinguish between the potential anxiolytic and attention-shifting effects of stress.

An alternative possibility is that release from the restraint of handling when the fish reached the test tank may have induced a relatively positive affective state. This is consistent with the idea that positive affect represents the differential of state across time, i.e. positive affect occurs not so much when things are good, but when they are getting better [48]. When the fish were released into the water following handling, they entered a more favourable environment. The more severe handling stress caused by net transfer may therefore have led to a bigger ‘release from restraint’ (‘relief’) effect, and a relatively more positive affective state. For example, Doyle et al. [49] observed that release from restraint resulted in sheep showing a more ‘optimistic’ cognitive bias consistent with positive affective response. Moreover, ‘relief-learning’ of cues which predict the offset of noxious stimuli is well established in a variety of vertebrate and invertebrate species, and appears to be positively-valenced [50].

Greater time spent higher in the diving/scototaxis tank may also have reflected an attempt to reach oxygen-rich waters close to the surface following removal from water during the net-handling procedure, and/or as a result of higher metabolic demands of the presumed stress state (e.g. higher gill beat rate) induced by net-handling [27]. However, this hypothesis does not explain the greater use of the white side of the tank following net transfer. A general escape-orientated response might also have resulted in a higher overall speed of movement. However, there was no difference in speed between the two handling conditions in the combined diving and scototaxis test and no main effect of handling on speed in the open-field and novel-object tests.

Finally, it is possible that the stress of net-handling caused these fish to swim more rapidly away from the release site (the centre of the top third of the black side of the tank) than box-handled fish at the start of the test. If so, we would expect differences in the locations of the two groups of fish to be more pronounced early on in the test but actually the opposite is the case (Fig. 4a–c). Moreover, the net-handled fish spent more time away from the lower third of the tank, and hence closer to the release site, than the box-handled fish.

The diving and scototaxis test yielded consistent differences in both of the key measures that this test is based on – depth in tank and time spent on the black vs white side – and also in a variable which integrates both measures; mean distance from the bottom dark area of the tank. Although there is some suggestion from pharmacological studies that, separately, the two tests may not measure exactly the same motivational or affective state (see Introduction, [19]), fish in our study showed a coherent response to both elements of the combined test indicating that the two test components were detecting a common affective process. If the two tests elements do detect subtly different aspects of anxiety, the combined test should provide a more sensitive measure than each test on its own, which can detect both aspects and which has both predictive and construct validity [19]. The combined test may also allow alternative interpretations to be discounted as in the present study where explanations of low anxiety-like behaviour in terms of motivation to reach higher oxygen-rich water do not account for the observed simultaneous greater use of the white side of the tank.

## 5 Conclusions

In conclusion, the lower levels of anxiety-related behaviour observed in net-handled fish in the combined diving and scototaxis test appear likely to have been a result of the short-term physiological stress of net-handling causing changes in attention or reward- and punishment-focused behaviour, and/or a relief-like state. Although these effects remain to be disentangled, for example in future studies that specifically measure attention, reward-seeking and affective valence, our findings indicate that handling techniques should be carefully considered when planning experiments involving fish. If the stress of net handling does interfere with normal cognitive and behavioural responses, then such interference could be a problem for any behavioural study where fish are handled in such a way. Our study also indicates that the combined diving and scototaxis test is able to reveal clear effects of handling treatments that are not reliably detected by conventional open-field and novel-object tests. This emphasises the utility of a simple test based on biologically meaningful measures of anxiety-like states.

## Figures and Tables

**Table 1:** Results of Mann-Whitney *U* tests of the effects of tank colour and handling method on behaviour in the Open Field and Novel Object tests.

*Note*: Italicised text indicates differences at p < 0.05 which were not significant following sequential Bonferonni correction. Grey cells indicate missing data (latency to enter central tank zone was not recorded in the open field test because fish were placed in the centre at the start of this test).

**Table 2:** Results of general linear model analyses for effects of time, handling and their interaction on measures from the combined diving and scototaxis test.

Measure	Data transformation	Handling main effect				Time main effect				Handling × time interaction effect			
F	df	p	partial-eta squared	F	df	p	partial-eta squared	F	df	p	partial-eta squared
Duration in bottom third of tank	None (x)	3.16	1,54	0.08	0.055	15.38	5.19,280.1	**<0.001**	0.222	2.70	5.19,280.1	**0.019**	0.048
Mean displacement from bottom dark area	Log_10_ (x + 1)	7.28	1,54	**0.009**	0.119	18.14	4.08,220.5	**<0.001**	0.252	0.83	4.08,220.5	0.51	0.015
Speed of movement	Log_10_ (x + 1)	0.11	1,54	0.74	0.002	73.34	5.11,276	**<0.001**	0.576	0.89	5.11,276	0.49	0.016

*Note*: Bold text indicates significant differences following sequential Bonferonni correction.
